# Aspirin and cancer: biological mechanisms and clinical outcomes

**DOI:** 10.1098/rsob.220124

**Published:** 2022-09-14

**Authors:** Peter Elwood, Majd Protty, Gareth Morgan, Janet Pickering, Christine Delon, John Watkins

**Affiliations:** ^1^ Division of Population Medicine, Cardiff University, Cardiff, Wales CF10 3AT, UK; ^2^ Department of Cardiology, Cardiff Lipidomic Group, Cardiff University, Cardiff, Wales, UK; ^3^ Public Health Wales, Cardiff, Wales, UK; ^4^ Independent Research, London, England, UK

**Keywords:** aspirin, cancer, survival, bleeding, thromboembolism

## Abstract

Evidence on aspirin and cancer comes from two main sources: (1) the effect of aspirin upon biological mechanisms in cancer, and (2) clinical studies of patients with cancer, some of whom take aspirin. A series of systematic literature searches identified published reports relevant to these two sources. The effects of aspirin upon biological mechanisms involved in cancer initiation and growth appear to generate reasonable expectations of effects upon the progress and mortality of cancer. Clinical evidence on aspirin appears overall to be favourable to the use of aspirin, but evidence from randomized trials is limited, and inconsistent. The main body of evidence comes from meta-analyses of observational studies of patients with a wide range of cancers, about 25% of whom were taking aspirin. Heterogeneity is large but, overall, aspirin is associated with increases in survival and reductions in metastatic spread and vascular complications of different cancers. It is important that evaluations of aspirin used as an adjunct cancer treatment are based upon all the available relevant evidence, and there appears to be a marked harmony between the effects of aspirin upon biological mechanisms and upon the clinical progress of cancer.

## 1. Introduction

The development of new drugs for the treatment of cancer is costly and time-consuming, and most of the drugs which pass laboratory testing fail in clinical trials and are not approved for use in clinical practice [1]. The concept of ‘old drug, new tricks' is leading to the testing of many approved drugs in the hope of extending the range of therapies in oncology [2].

The use of natural products has been a successful strategy in the discovery of medicines of possible value in the treatment of cancer [3]. Hence our interest in acetyl salicylic acid (aspirin), the main part of which is widespread and is highly active within plants. Many years of botanical research has established salicylates as a potent hormone governing various responses to abiotic and biotic stress, a major phytohormone influencing defence in plants against a wide variety of pathogens and a regulator of programmed cell death—activities which are likely to be of clinical relevance in human patients (L. Mur 2021, personal communication).

One in every six deaths worldwide is due to cancer [4], giving an estimated 9.6 million in 2018, with around 70% of the deaths in low- and middle-income countries [5]. The World Health Organization points out that most cancers in poorer countries are diagnosed at a very late stage, when most treatments are no longer effective, even if treatments were available (which they are not in many countries) [6].

In this review, we summarize the findings on mechanisms by which aspirin can affect the pathogenic pathways of neoplastic processes at a cellular level, and the influence of aspirin on the dynamics of metastatic cancer spread. We then summarize the evidence on survival in clinical studies of aspirin taking in randomized trials and in observational studies, and the effect of aspirin on metastatic cancer spread and on thromboembolic complications of cancer. Finally, we address the safety aspects of aspirin use in relation to the risks of an increase in gastrointestinal and intra-cranial bleeding attributable to aspirin.

## 2. Aspirin and biological mechanisms relevant to cancer

In this section, we summarize the biological mechanisms of aspirin which are relevant in cancer pathogenesis. Specifically, the main mechanism of aspirin, its impact on cancer pathways, on proliferation and on metastasis, thrombosis and DNA repair.

### 2.1. Mechanisms and anti-cancer pathways

The primary mechanism of aspirin is disruption of the cyclooxygenase (COX) enzyme responsible for the formation of key signalling lipids known as prostanoids. While this is an important pathway in cancer signalling, recent evidence highlights additional targets for aspirin in tackling cancer progression [7,8]. Using human breast and ovarian cancer cell lines, aspirin has been shown to beneficially interfere with energy metabolism by targeting key enzymes involved in the proliferation of cancer cells both directly and through COX inhibition [9,10]. In addition, using *in-vitro* enzymatic assays and human cancer cell line studies, aspirin inhibited cancer progression through interference with proliferative pathways [11], cancer-associated inflammation [12] and platelet-driven pro-carcinogenic activity [13].

### 2.2. Angiogenesis and aspirin

Moreover, cancers rely on angiogenesis in order to grow and spread. In studies on human colon cancer and lymphoma cell lines, aspirin appeared to have a direct impact on angiogenesis by both inhibiting the COX enzyme which is frequently overexpressed in cancer cells, as well as by directly modulating vascular-endothelial growth factor (VEGF) activity [14,15]. Other beneficial effects of aspirin include stimulation of pro-apoptotic pathways [16] and the enhancing of p53 mediated DNA repair [17] as demonstrated in studies using human breast and colon cancer cell lines.

### 2.3. Aspirin in metastatic spread

The first evidence of benefit from aspirin in cancer, was the demonstration almost 60 years ago of a reduction in metastatic cancer spread in animal models *in vivo* [18] which was related to the anti-platelet effect of aspirin. Many authors have since confirmed the association in a range of cancers [19,20]. In studies using *in vivo* animal models of metastasis, and *in vitro* models of cell invasion with human cell lines, platelets were shown to play a significant role in metastasis via a number of mechanisms [20,21], including secretion of growth factors which enable metastatic migration [22,23], association of platelet aggregation with tumour cells promoting early metastatic niche formation, secretion of microDNA inhibitors of tumour suppressor genes [22,24], and interfering with phospholipid metabolism leading to the formation of pro-metastatic signals [25]. It is logical therefore that inhibiting platelet function will serve as an effective anti-metastatic treatment [21].

### 2.4. Evidence for aspirin in cancer-related thrombosis

Patients with cancer appear to be in a hypercoagulable state [26,27] with marked increases in vascular [28] and thromboembolic disease events [29]. While aspirin is not considered to be anti-coagulant it has been shown to reduce thromboembolism [30,31], including in patients with cancer [32], probably by blocking COX and inhibiting the formation of thromboxane A2 (TxA2), a potent driver of thrombosis in vascular disease [33]. TxA2 has in fact been shown to be elevated in some cancers and this may contribute to the raised thromboembolic risk in cancer, as described above [21,34–36].

### 2.5. Mendelian randomization and benefit of aspirin in cancer

A completely different approach to the evaluation of aspirin comes from quasi-randomized studies based upon gene/environmental interaction, or ‘Mendelian randomization’. The logical basis of this is that genetic variants avoids confounding and yield evidence similar to that of randomized trial [37]. A polymorphism that changes a nucleotide in COX-2 leads to effects that mimic some of the biological effects of aspirin [38,39]. This was observed in case-control studies in African-Americans with the polymorphism, suggesting a reduction in colorectal adenomas (odds ratio, OR 0.56; 95% CI 0.25, 1.27) in 61 patients with the polymorphism, and in another study there was a possible reduction in colorectal cancer in 138 patients (OR 0.67; 0.28, 1.56) [39].

### 2.6. Lynch syndrome and aspirin

Many of the biological effects that are described above that are exerted by aspirin suggest that benefit from the use of this drug in cancer is a reasonable expectation, though to date the drug appears to be recommended only in the UK and only for patients with Lynch syndrome [40]—a rare dominant genetic error associated with a high risk of colon and other cancers.

Work on Lynch syndrome shows the mismatch repair of DNA to be mechanism protective against cancer [38,39] and its failure could occur in any of us. Hence aspirin, through its enhancement of this mechanism [41] would appear to be a potential prophylactic within all of us. This last is echoed in a recent study by Nounu *et al.* [42] which combined proteomics and Mendelial randomization to highlight a link between levels of proteins involved in DNA repair which are affected by aspirin supplementation and cancer incidence. Specifically, aspirin was observed to reduce the expression of MCM6 and RRM2, both involved in DNA repair, in human colon cells. The study followed this observation with a Mendelian randomization analysis of a large case-control cohort which showed that increases in the protein/mRNA expression of these two proteins was associated with increased colon cancer risk (OR 1.08; 1.03, 1.13; and OR 3.33; 2.46, 4.50, respectively) [42], concluding that the beneficial effect of aspirin in cancer many be through enhancement of DNA repair mechanisms.

## 3. Clinical effects associated with aspirin

### 3.1. Randomized trials of aspirin and mortality

Systematic literature searches identified four early *ad hoc* randomized trials of aspirin and cancer treatment [43–46]. The pooling of the results of these gives a suggestive reduction of 9% in cancer deaths in the 722 in patients with cancer who had been randomized to aspirin (HR 0.91; 95% CI 0.79, 1.04).

During the US Physicians Health Study of aspirin and cancer prevention, an opportunistic trial was conducted in 502 subjects who having been randomized to take aspirin, developed cancer of the prostate. These were followed and a 30% relative reduction was attributable to aspirin (HR 0.68, 95% CI 0.52, 0.90 in cancer deaths and HR 0.72; 0.61, 0.9 in all-cause deaths) [47].

Earlier this year a report of 3021patients with a HER2-negative breast cancer who had been randomized to 300 mg aspirin daily was reported. During a median follow-up of 20 months 191 invasive events had occurred (84 on aspirin 107 on placebo 84: HR 1.27) [48]. Even more recently 95 patients with locally advanced metastatic gastric cancer were randomized, 45 to receive 150 mg aspirin daily, 45 to receive no aspirin [49]. After a median follow-up of 29 months the median survival of patients on aspirin was 10 months compared with 11 months in patients who received no aspirin (*p* = 0.90).

During 2007–2012, a series of long-term follow-up studies were conducted in subjects who had already participated in earlier British and European randomized trials of aspirin and vascular disease reduction [19,50]. The development of cancer in subjects who had been involved in more than 50 randomized vascular trials were followed-up for up to 20 years. The studies focused mainly upon incident cancer but within several of the studies there is evidence consistent with a reduction in cancer mortality. On this last, for example—OR 0.58 (95% CI 0.44, 0.78) in an overview of six randomized trials [19], and OR 0.84 (96% CI 0.75, 0.94) for cancer deaths in an overview of 51 randomized trials [50].

### 3.2. Observational studies of aspirin and mortality

The bulk of the evidence on aspirin as an adjunct treatment comes however from observational cohort and case-control studies of patients with cancer, some of whom (usually about 25%) were taking aspirin, most often for vascular disease prevention. These enabled the testing of the hypothesis that aspirin reduces mortality across a range of different cancers. A series of three replicate systematic literature searches followed by meta-analyses yielded closely similar estimates of the reduction in cancer mortality associated with aspirin: in 2016 a pooled hazard ratio (HR) for eight different cancers (overall HR 0.85; 95% CI 0.77, 0.92) [51], in 2018 based on 10 different cancers (HR 0.74; 0.66, 0.82) [52] and in 2021 based on 18 different cancers (HR 0.78; 0.67, 0.91) [53].

In the report in 2020 [53], the hypothesis that the benefits of aspirin in the treatment of cancer are relevant to a wide range of cancers was tested in detail in 118 reports based on 18 different cancers. An overall pooled reduction in cancer mortality of about 20% was associated with aspirin taking (HR 0.77; 95% confidence limits 0.72, 0.83 in 70 papers which reported effects as HRs, and OR 0.67 (0.45, 1.00 in 11 reports that used this measure of association). All-cause mortality showed closely similar reductions: HR 0.79 (0.74, 0.86) in 56 papers that used hazard ratios, and OR 0.57 (0.36, 0.89) in seven papers that reported odds ratios.

An important finding in this report [53] is that a meta-analysis of the results reported for aspirin in 39 publications focused upon 15 less common cancers (nasopharyngeal, oropharyngeal, oesophagus, gastrointestinal, gastric, rectal, liver, gallbladder, bladder, pancreas, bladder, endometrium, ovary, glioma, head and neck, lung, melanoma) reported overall reductions associated with aspirin (HR 0.79; 0.70, 0.88 in 18 studies, and OR 0.49; 0.26, 0.95 in 5 studies). These reductions are comparable to the reduction in colon cancer, the cancer in which the effect of aspirin has been examined most frequently (HR 0.71; 0.62, 0.80 in 24 studies, and OR 0.78; 0.66, 0.93 in one study).

Publication bias in this study was examined in detail [53]. The judgement of the authors was that while conclusions drawn from these 118 reports, not having been randomized, have to be accepted with caution. However, the evidence is strengthened by the absence of significant bias at *p* < 0.05 for the data for colon cancer alone, or all cancer combined. Furthermore, an exacting test for publication bias—a ‘trim and fill’ analysis on the data for the 39 less common cancers—reduced, but maintained the statistical significance of, the beneficial treatment effect for both cancer mortality and all-cause mortality.

### 3.3. Aspirin and duration of cancer survival

A few authors have made estimates of the length of additional survival associated with aspirin taking. A number of different summary statistics of survival have been used, and these defy pooling, but they are listed elsewhere [53] and they range from about three months up to three years [54]. Using a different approach, a group in Liverpool extracted extensive baseline data, including aspirin taking, from the records for 44 000 patients with colon cancer. With these they constructed a formula giving predicted estimates of survival [55]. Entering the details for a typical non-diabetic subject aged 70 with colon cancer into the formula, the inclusion of aspirin increases the estimate of survival by about five years for a man, and for a woman, about 4 years.

### 3.4. Aspirin and metastatic cancer spread

The effect of aspirin on mechanisms involved in metastatic spread is of particular importance because metastases are responsible for much of the pain and the complications of cancer, [56] and many of the deaths are attributable to the metastases rather than to the primary tumour itself [57]. In trials and observational studies of a range of different cancers a reduction in metastatic spread associated with aspirin is shown. Thus, in a review of five randomized trials there was a 35% reduction in distant metastases associated with aspirin taking (HR 0.64, 95% CI 0.48 to 0.84) [58]. Another review reported an RR of 0.48 (0.30, 0.75) in three randomized studies and 0.69 (0.57, 0.83) in five observational studies [19]. Yet another pooled estimate in five reports gave the reduction in metastatic spread associated with aspirin as RR 0.77 (0.65, 0.92) [51]. An effect on cancer spread suggests that the drug has value in the treatment of cancer independent of any effect on mortality.

### 3.5. Aspirin and thromboembolic complications of cancer

Thromboembolism is a serious complication of cancer, to which aspirin is known to be of relevance. The Surveillance, Epidemiology and End Results (SEER) programme on mortality in cancer patients reported that 11% of deaths amongst 20 cancer types had been certified as due to vascular disease, most of which (76%) was heart disease [28]. Vascular mortality was particularly high within the first year after diagnosis of cancer and remained high thereafter.

A recent report of thromboembolism in UK patients with cancer was introduced with the comment that the substantial improvements in cancer survival during the past few decades has led to increasing concerns about long-term cardiovascular risks in cancer survivors [29]. The authors therefore examined relevant records for 108 000 survivors of a range of cancers. Venous thromboembolism was found to be substantially elevated in patients with almost all the cancers (SMR 3.93; 3.89, 3.87) and although the risk fell over time it remained elevated more than five years after the diagnosis of cancer. Coronary artery disease and stroke were increased as well as heart failure, cardiomyopathy and other vascular disease events.

Venous thromboembolic disease is one of the leading causes of death in cancer [28] and is reduced by aspirin, [32] leading the American Society of Clinical Oncology to recommend that prophylactic anticoagulants be considered for all hospitalized cancer patients [59,60]. Although aspirin use has to some extent been superseded by recently developed drugs for vascular protection, aspirin has been shown to be effective against vascular and venous thrombosis [31,32].

Putting all the above together, aspirin appears to affect some of the biological mechanisms relevant to cancer favourably, and, taken together, the clinical evidence appears to be supportive of an increase in cancer survival, a reduction in metastatic spread and a reduction in cancer-related vascular mortality. Questions however arise about the safety, and the risk-benefit balance of aspirin in cancer.

## 4. Adverse effects of aspirin

A bleed, either gastrointestinal or intra-cerebral, is a crisis for a patient and especially for patients who are already seriously ill. Yet the seriousness of bleeds attributable to aspirin, and not just their frequency, should be evaluated against the benefits which may be attributable to its use [61,62].

### 4.1. Gastrointestinal bleeding and aspirin

Low-dose aspirin is associated with additional gastrointestinal (GI) bleeds in between 0.8 and 5.0 patients per 1000 person years aged 50–84 years in the general population. This represents an increase above spontaneous GI bleeding of between about 50% [63] and 90% [64]. It is important to note that these increases imply that only one in every two or every three bleeds that occur in patients taking low-dose aspirin is likely to be truly attributable to the aspirin, the other bleeds being spontaneous and not directly due to aspirin.

### 4.2. Fatal GI bleeds and aspirin

Almost every published report limits bleeding to events termed ‘serious’, ‘major’ or ‘hospitalized’—terms which are based on rather vague value judgements. The most serious bleeds are those that lead to death—a dichotomy with certainty! A systematic search identified 11 randomized trials, comprising a total of over a 100 000 patients, half of whom had been randomized to aspirin [65]. The table shows that the expected risk of a ‘major’ bleed for aspirin was confirmed, but the relative risk of a fatal bleed in patients taking aspirin was substantially reduced (relative risk compared with spontaneous bleeds in subjects randomized to placebo: RR 0.45; 0.25, 0.80; table 1).

**Table 1.** GI bleeding in a meta-analysis of data from 11 trials in which aspirin had been randomized [62].

bleeding	risk per year	relative risk for aspirin
incidence of a GI bleed		
in 54 625 subjects randomized to aspirin	8 per1000	RR 1.55
in 52 583 subjects randomized to placebo	5 per 1000	(1.32, 1.83)
proportion of bleeds that were fatal		
in subjects on aspirin	4%	RR = 0.45
in subjects on placebo	8%	(0.25, 0,80)
risk of a fatal bleed in trial participants		
randomized to aspirin	3.7/10 000	RR = 0.77
randomized to placebo	4.7/10 000	(0.41, 1.43)

Other studies have shown similar reductions for fatal bleeding and aspirin [66–69], and it is of interest that in the recent ASPREE trial of almost 20 000 subjects, only two fatal bleeds occurred—both in patients randomized to placebo [70]. It has been suggested that aspirin may unmask existing gastrointestinal pathology and precipitate bleeding at a relatively early stage of the development of the pathology responsible for the bleed, when it can be more easily and more successfully treated. A US Task Force made a similar interpretation: that the predominant adverse anti-platelet effect of aspirin is to promote bleeding from established pathological gastric lesions [71].

### 4.3. Intra-cerebral bleeding and aspirin

The risk of cerebral bleeding is also increased by aspirin, again by around 50%, this being equivalent to one or two events per 10 000 subjects per year [63,72]. A number of authors comment on the probable reduction in cerebral bleeding if blood pressure is measured before aspirin is started, and hypertension, if present, is adequately treated [72]. Evidence which strongly supports this comes from a randomized trial based on more than 18 000 hypertensive patients, all of whom were receiving ‘optimal’ antihypertensive treatment. Among almost 10 000 patients randomized to aspirin there were seven fatal bleeds, and in 10 000 patients randomized to placebo there were eight fatal bleeds [73].

## 5. Discussion

Evidence of benefit from aspirin comes from many sources: its effects upon biological mechanisms relevant to cancer; the results of a few randomized trials and outcome data from many observational studies. Furthermore, the biological mechanisms leading to reductions in metastatic spread and in thromboembolic complications of cancer are a consequence of biological effects of aspirin different to, and probably independent of the mechanisms relevant to mortality. Aspirin could therefore have value in the treatment and care of patients, independent of its effects on mortality.

Heterogeneity is to be expected in overviews of such a wide field of clinical activity as this but one is surprised by the degree of heterogeneity shown in the published studies of aspirin and survival. Further uncertainties on aspirin arise from major trials, such as the US Women's Health Study [74] and the Australian ASPREE trial [75,76], neither of which detected benefit from aspirin. One wonders if there are powerful, but as yet unidentified factors confounding relationships with aspirin. Further work on this should be fruitful.

The lack of adequate *ad hoc* randomized trials on aspirin treatment of a wide range of cancers is serious limitation in the available evidence. Furthermore, almost all the current research appears to focus upon common cancers: colon [77–79], breast [77], prostate [77,80] and lung cancer [81]. The three cancers account for only about 30% of the worldwide cancer burden and studies focused upon them will add little to the problem of the less common cancers, including the ‘rare’ cancers (usually defined as those with an incidence of less than 6 per 1 000 000), which account for about 22% of all cancers and have a worse survival (47%) than the common cancers (65%) [82].

Clearly, further evidence is needed and it would seem to be unreasonable to require randomized evidence for every separate cancer. Furthermore, even if one waits for new randomized evidence, the validity of one or two randomized trials as a basis for recommendations is limited and will truly relate to only the cancer or cancers actually trialled. There is therefore a desperate need for more evidence on aspirin use in the less frequent cancers.

Findings in ASPREE, a trial of cancer prevention by 100 mg aspirin daily in older, healthy subjects are a serious challenge to the use of aspirin [75,76]. With an average follow-up of only 4.7 years, cancer-related deaths occurred in 3.1% of the ten thousand participants randomized to aspirin, and in 2.3% of the 10 000 randomized to receive placebo (HR 1.34; 95% CI, 1.10–1.56). This led the authors to suggest a possible adverse effect of aspirin on cancer evolution in older adults. Clearly this finding is unique to this prevention study, and further evidence from a much longer follow-up is urgently required. Furthermore, compliance with tablet taking, which was monitored throughout ASPREE had fallen by 40% at the time of the follow-up [75,76].

This last—compliance with treatment—is a major uncertainty in observational studies, many of which are conducted retrospectively. A group in Dublin monitored compliance in detail and they detected an influence of approaching death on aspirin use in patients with breast and with colorectal cancer. The taking of aspirin declined ‘considerably’ during the 2 years before death, and at the time of death, rates of aspirin use had dropped from around 60% to around 20% in patients with colorectal cancer and from around 80% to around 45% in patients with breast cancer [83].

On the other hand, discontinuation is of importance, because the sudden stopping of aspirin has been shown to increase vascular disease events—up to threefold increases in major cardiac events [84,85] and a similar increase in ischaemic strokes [86].

## 6. Conclusion

There appears to be an impressive harmony between the biological effects of aspirin on mechanisms relevant to cancer, and the effects of aspirin on clinical outcomes in cancer. Further evidence is needed before the suggestion by the present evidence of about a 20% increase in survival of caner is accepted, as it is to be hoped that research will eventually explain some of the large heterogeneity in the present evidence. Fortunately, research on aspirin taken by cancer patients can be conducted with a high degree of confidence that aspirin is a relatively safe drug.

## Data Availability

This article does not contain any additional data.
